# Regulation of senescence escape by the cdk4–EZH2–AP2M1 pathway in response to chemotherapy

**DOI:** 10.1038/s41419-017-0209-y

**Published:** 2018-02-07

**Authors:** Mélanie Le Duff, Julien Gouju, Barbara Jonchère, Jordan Guillon, Bertrand Toutain, Alice Boissard, Cécile Henry, Catherine Guette, Eric Lelièvre, Olivier Coqueret

**Affiliations:** Paul Papin ICO Cancer Center, CRCINA, INSERM, Université d’Angers, Angers, France

## Abstract

Senescence is a tumor suppressive mechanism that induces a permanent proliferative arrest in response to an oncogenic insult or to the genotoxic stress induced by chemotherapy. We have recently described that some cells can escape this arrest, either because senescence was incomplete or as a consequence of a phenotypic adaptation. Malignant cells which resisted senescence emerged as more transformed cells that resist anoikis and rely on survival pathways activated by Akt and Mcl-1. In this study, we further characterize senescence escape, investigating how emergent cells could reproliferate. During the initial step of chemotherapy-induced senescence (CIS), we found that cyclin D1 was upregulated and that cell emergence was prevented when its main partner cdk4 was inactivated. Results indicate that this kinase induced the upregulation of the EZH2 methylase, a component of the polycomb PRC2 complex. Downregulated during the early step of treatment, the methylase was reactivated in clones that escaped senescence. The inactivation of EZH2, either by siRNA or by specific inhibitors, led to a specific inhibition of cell emergence. We used quantitative proteomic analysis to identify new targets of the methylase involved in senescence escape. We identified proteins involved in receptor endocytosis and described new functions for the AP2M1 protein in the control of chemotherapy-mediated senescence. Our results indicate that AP2M1 is involved in the transmission of secreted signals produced by senescent cells, suggesting that this pathway might regulate specific receptors involved in the control of CIS escape. In light of these results, we therefore propose that the cdk4–EZH2–AP2M1 pathway plays an important role during chemotherapy resistance and senescence escape. Since targeted therapies are available against these proteins, we propose that they should be tested in the treatment of colorectal or breast cancers that become resistant to first-line genotoxic therapies.

## Introduction

It is now well accepted that senescence plays a critical role in the suppression of tumorigenesis and in the response to chemotherapy, both in vitro and in vivo^1^. This implies that senescence bypass is a key feature of tumor progression, either during the early stages of carcinogenesis or during treatment failure. However, since senescence is theoretically irreversible, it is not really clear how this escape can take place^2^. Accumulating studies using different experimental models suggest that this suppressive mechanism can be reversed. In fibroblasts, replicative senescence relies on the induction of p53–p21 but maintaining this arrest depends on the presence of p16INK4^3^. For instance, phosphatase and tensin homolog (PTEN) depletion reverses established senescence induced by the BRAF oncogene and this leads to tumor progression^4^. In colorectal cancer, we have recently described two models of senescence escape, in response to oncogene^5,6^ or during chemotherapy-induced senescence (CIS)^7,8^. In both cases, we have observed that a subpopulation of cells escapes this arrest and emerges as a more aggressive, dividing population. Cells that resist CIS grow in low adhesion conditions, form tumors in vivo and rely on Akt-Mcl-1 signaling. In this experimental model, we concluded that the coexistence of senescent and dividing subclones favored cell emergence in response to chemotherapy. We have therefore proposed that apoptosis is a superior suppressive mechanism as compared to CIS, at least in response to irinotecan.

In the current study, we pursued these experiments on the characterization of CIS escape, with the aim of understanding how emergent cells could reproliferate and finding combination therapies that could prevent division. Despite the fact that cyclin D1 is essentially known as an activator of the G1 phase of the cell cycle^9^, we describe in this work that this protein is significantly upregulated during the initial step of chemotherapy-mediated senescence. The inactivation of cdk4 significantly enhanced treatment efficacy and prevented cell emergence, indicating that this kinase plays an important role in CIS escape. This effect was correlated with the upregulation of the EZH2 protein, a histone H3K27 methylase activated by E2F signaling. Our results indicate that the cdk4 pathway upregulated EZH2 to induce cell emergence and that the inactivation of the methylase prevented CIS escape. Quantitative proteomic analysis allowed us to identify new targets of EZH2 involved in emergence, and we described new functions for the AP2M1 protein, initially known to be involved in receptor endocytosis^10^.

Therefore, although chemotherapy killed the vast majority of the initial population, some cells escape chemotherapy-mediated senescence and reproliferate due to the activation of the cdk4–EZH2 pathway. We propose that targeted therapies against this signaling should be considered to reduce emergence and improve the treatment of colorectal or breast cancers resistant to first-line therapies.

## Results

### Subpopulations of cells escape chemotherapy-mediated senescence

**Fig. 1:**
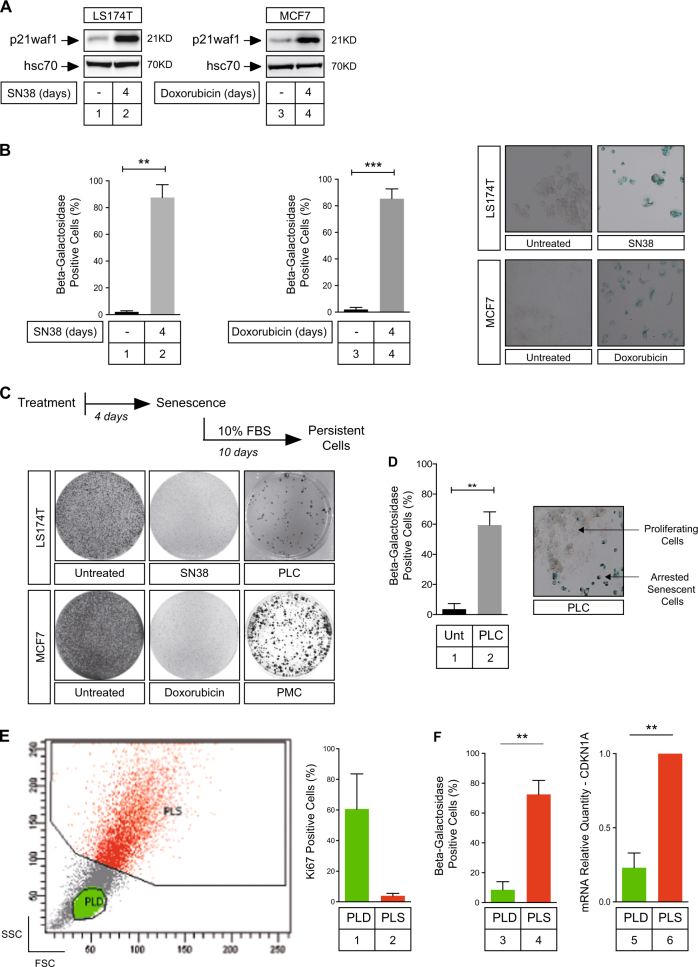
Colorectal and breast cancer cell lines escape chemotherapy-mediated senescence. **a**,** b** LS174T cells have been stimulated or not with sn38 (5 ng/ml) for 4 days as indicated. MCF7 cells have been treated or not with doxorubicin (15 ng/ml). Senescent cells were evaluated by the detection of p21waf1 expression by western blot (*n* = 6 for LS174T cells, 5 for MCF7 cells) and by SA-β-galactosidase staining (*n* = 6 for LS174T cells, 7 for MCF7 cells; **p < 0.01; ***p < 0.001). **c** Cells were treated as above for 4 days and were stimulated with 10% FBS for 10 days. Crystal violet coloration was used to visualize emergent clones, called PLC (persistent LS174T cells) or PMC (persistent MCF7 cells); *n* = 10 for LS174T, 6 for MCF7 cells. **d** SA-β-galactosidase activity was analyzed in PLC (*n* = 5; **p < 0.01). Note the emergence of dividing clones in the middle of arrested cells. **e** Emergent PLC cells were analyzed by flow cytometry after Ki67 staining, cells have been gated according to low (green) or high (red) FSC/SSC values and the corresponding Ki67 plots are shown (*n* = 3). The PLC population is heterogeneous and composed of around 60–70% senescent cells named PLS and 30–40% of proliferating cells named PLD. **f** Emergent cells were sorted by flow cytometry according to low (PLD) or high (PLS) FSC/SSC values and SA-β-galactosidase activity was analyzed in each subpopulation (*n* = 6; **p < 0.01). The mRNA expression of the CDKN1A gene (p21waf1) has been evaluated by quantitative RT-PCR in the dividing PLD and senescent PLS subpopulations (*n* = 6; **p < 0.01)

We have recently described that subpopulations of colorectal cells can escape CIS and resume proliferation^7,8,11^. This was confirmed in this study using either colorectal cut at colo-rectal with the pdf margin instead of color-ectal (LS174T) or breast (MCF7) cancer cells that entered senescence when treated respectively with sn38 or doxorubicin. We initially described this suppressive arrest using clonogenic tests and heterochromatin foci^7,8,12^; it is shown here by detecting β-galactosidase staining and p21waf1 expression (Fig. 1a, b). Senescence escape leads to the emergence of more transformed cells that we have named PLC (persistent LS174T cells) and PMC (persistent LS174T replace LS174T by MCF7 cells, Fig. 1c). These emergent cells are more aggressive than parental cells since they grow in low adhesion conditions and are resistant to anoikis^7,8^. We have previously shown that senescence escape is not explained by the presence of a resistant clone within the parental population^7,8^. To confirm this observation, the activation of cell cycle genes was investigated following senescence induction (Supplementary Figure 1A). No significant induction of S-phase genes such as mcm2, mcm3, cdc25A or cyclin A was noticed following serum stimulation of arrested cells. As a control, these genes were activated following activation of quiescent cells (Supplementary Figure 1B and C). We also analyzed cell division during the acute induction of senescence. To this end, cells were seeded in 96 wells per plate at a concentration of one to four cells per well. Clones were then grown in 3% fetal bovine serum (FBS) or treated with sn38 for 6 days. A resistant clone would proliferate to the same extent as non-treated parental cells in these conditions. However, dividing cells were not detected, further indicating that senescence escape was not due to the presence of a resistant clone in the parental population (Supplementary Figure 1D). After 7 days, the emergent PLC population is heterogeneous and composed of around 60–70% senescent cells (named PLS (persistent LS174T senescent) cells) and 30–40% of proliferating cells (named PLD (persistent LS174T dividing) cells). The presence of proliferating subclones, in the middle of arrested cells, is shown using negative β-galactosidase staining (Fig. 1d). Using flow cytometry analysis, we have previously described that we can identify the dividing PLD subpopulation within emergent cells according to a low forward-scatter (FSC) and side-scatter (SSC) profile and a high Ki67 staining^7,8^. FSC^low^/SSC^low^ PLD cells were associated with a high Ki67 staining indicative of cell division. On the contrary, PLS cells that remained senescent (FSC^high^/SSC^high^) express p21waf1, stained positive for β-galactosidase and have low levels of Ki67 (Fig. 1e, f).

### Cyclin D1 is upregulated during the initial step of senescence induction

**Fig. 2:**
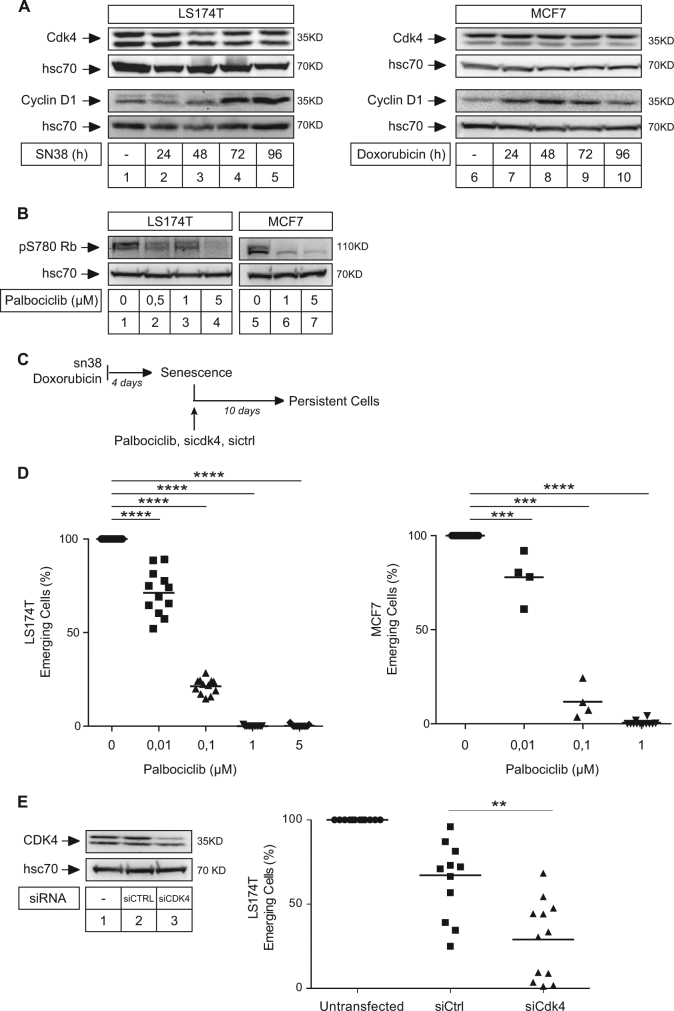
cdk4 is necessary to induce CIS escape. on the pdf it's written figure on the previous page whereas it should be next page. The same on the bottom of figure 2, it's written next instead of previous) **a** LS174T cells and MCF7 cells have been stimulated with sn38 (5 ng/ml) or doxorubicin (15 ng/ml) respectively. Cdk4 and Cyclin D1 expressions were analyzed by western blot (*n* = 6 for LS174T cells and 5 for MCF7 cells). **b** LS174T and MCF7 cells have been stimulated with Palbociclib for 48 h, phosphorylation of Rb at the S780 site was evaluated by western blot (*n* = 5). **c** After treatment, cells were washed and stimulated with 10% FBS for 10 days to reinduce PLC emergence, in the presence or not of palbociclib or following transfection with siRNAs. **d** Crystal violet coloration was performed after 10 days and the quantification of emergent clones is presented in LS174T and MCF7 cells (*n* = 12 for LS174T cells and *n* = 4 for MCF7 cells; ***p < 0.001; ****p < 0.0001). **e** Cdk4 expression was analyzed by western blot 3 days after siRNA transfection (*n* = 4). Quantification of emergent clones is presented in LS174T cells (**p < 0.01)

We have previously shown that LS174T cells are arrested with a 4N DNA content in response to treatment^7,8^. To understand how emergent cells could reproliferate, we analyzed the expression of cyclin D1 and cdk4 since these proteins are the main regulators of the transition between G2/M and the next G1 phase. Western blot analysis showed that cdk4 level was not modified but a significant increase in cyclin D1 expression was observed, both in colorectal or breast cells (Fig. 2a, lanes 1–5 and 6–10). Reverse transcription quantitative PCR (RT-QPCR) experiments indicated that its messenger RNA (mRNA) was upregulated during the initial step of senescence induction in LS174T cells (Supplementary Figure 2A). We used chromatin immunoprecipitation experiments (ChIP) to characterize the regulation of its promoter. Whereas classical activators of cyclin D1 such as nuclear factor (NF)-κB2, STAT3 (signal transducer and activator of transcription 3) or EGFR (epidermal growth factor receptor) were not detected, results showed that E2F1 was associated with this gene. The type II RNA polymerase was also detected, mainly on the proxymal promoter (Supplementary Figure 2C). Surprisingly, its binding was not increased in response to treatment. As a control, its recruitment to the p21waf1 promoter was induced as expected (Supplementary Figure 2D). Using primers located within the exons 2 and 5 and intron 2, we observed that the binding of the elongating form of the polymerase was also not increased following treatment. These results suggested that the cyclin D1 gene was not regulated at the transcriptional level in response to treatment.

Since cdk4 is the main partner of cyclin D1, we then asked if the kinase was implicated in CIS escape. We first used palbociclib, a specific ATP-competitive inhibitor of cdk4^13^. As expected, palbociclib effectively blocked Rb-S780 phosphorylation, the main target site of the kinase (Fig. 2b). To analyze the effect of palbociclib on emergence, we added the drug at the time of serum release (Fig. 2c). The results presented in Fig. 2d indicated that palbociclib significantly limited CIS escape. This was observed in both LS174T and MCF7 cells. Note that if this drug was added at the same time as sn38 or doxorubicin, it prevented cell cycle progression and through a nonspecific effect blocked the effect of these drugs (data not shown). To confirm this observation, we then used RNA interference to downregulate cdk4 expression in LS174T cells. As expected, cdk4 levels were reduced as compared to cells transfected with a control small interfering RNA (siRNA) (Fig. 2e, left). To determine if the kinase was involved in CIS escape, persistent cells were generated following its inactivation. As shown in Fig. 2e, cdk4 downregulation significantly decreased the percentage of emerging clones. The same effect was observed in MCF7 cells (data not shown).

We concluded from these results that cdk4 was necessary for cell emergence in response to genotoxic treatment.

### The EZH2 methylase is activated by cdk4 and reexpressed in emergent clones

**Fig. 3:**
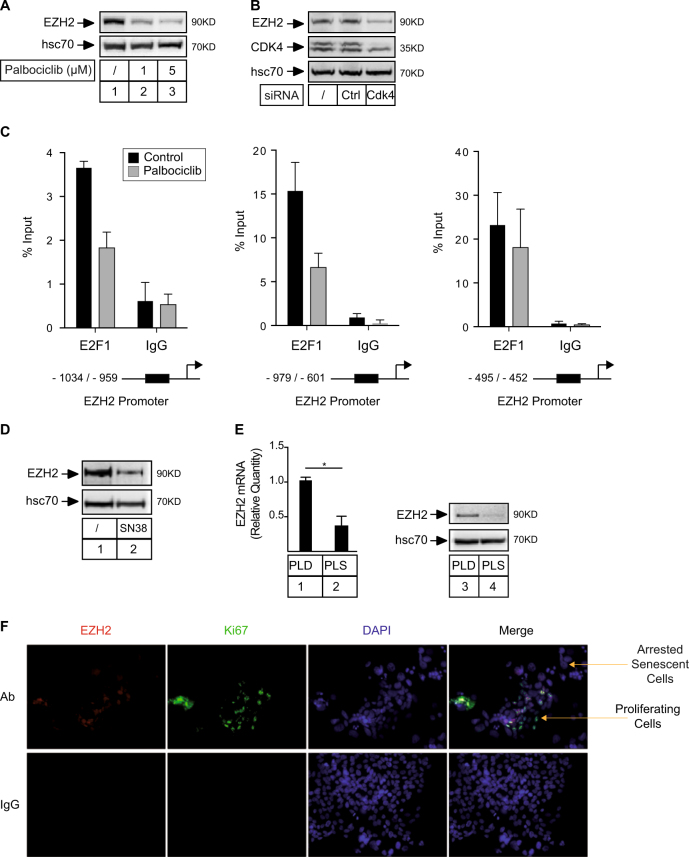
EZH2 is reactivated in emergent clones. **a** LS174T cells have been stimulated with the indicated concentrations of Palbociclib for 48 h. EZH2 expression was analyzed by western blot (*n* = 3). **b** LS174T cells have been transfected with the indicated siRNAs for 48 h. EZH2 expression was analyzed by western blot (*n* = 3). **c** LS174T cells have been stimulated with Palbociclib for 48 h. ChIP assays have been performed using the indicated primers and analyzed by quantitative PCR (*n* = 3). **d** Cells have been treated with sn38 (5 ng/ml) for 4 days. EZH2 expression was evaluated by western blot analysis (*n* = 4). **e** Following cell sorting, EZH2 expression has been evaluated by quantitative RT-PCR and by western blot in the dividing PLD and senescent PLS subpopulations (*n* = 6; **p < 0.01; n=4, *p < 0.05). **f** Analysis of EZH2 and Ki67 expression by immunofluorescence in PLC emergent cells (*n* = 4)

Since Cdk4 is a well-known activator of E2F, we then determined if CIS escape was related to the activation of its target genes. We focused on the EZH2 methylase since it is regulated by E2F and it plays an important role in tumor progression^14–16^. We first confirmed that EZH2 was a cdk4 target in our experimental conditions. To this end, cells were treated with palbociclib and its expression was evaluated by western blot experiments. As expected, the methylase was downregulated (Fig. 3a). The same result was observed when the kinase was downregulated by RNA interference (Fig. 3b). Chromatin immunoprecipitation experiments were used to confirm that E2F1 was present on the proxymal promoter of EZH2 (Fig. 3c).

We then analyzed the expression of EZH2 in response to treatment and during CIS escape. Western blot experiments indicated that the methylase was downregulated during the acute response to treatment (Fig. 3d). Using cell sorting following emergence, we observed that EZH2 was reactivated in the PLD dividing clones as compared to PLS cells that remained senescent, both at the protein and mRNA levels (Fig. 3e). To confirm that EZH2 was upregulated in dividing clones, we used immunofluorescence and Ki67 or EZH2 staining. Cells were treated as described above and senescence escape was induced for 7 days. Immunofluorescence was performed on emergent cells and dividing clones were identified by a positive Ki67 signal. Interestingly, results showed that EZH2 staining was mainly detected in cells that expressed the Ki67 proliferative antigen (Fig. 3f).

Altogether, we concluded from these results that EZH2 is downregulated during the early step of senescence and then reexpressed in the dividing subpopulation that restart proliferation.

### EZH2 allows cell emergence

**Fig. 4:**
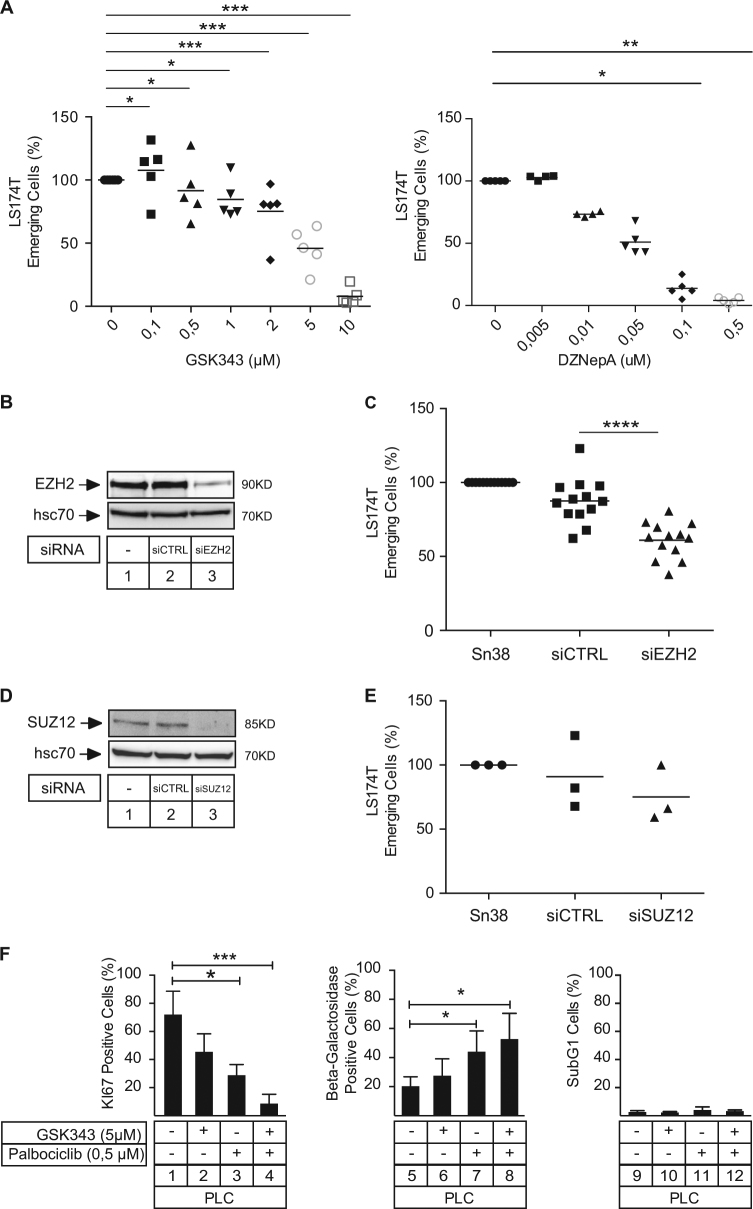
EZH2 is necessary during CIS escape. **a** LS174T cells have been stimulated with sn38 and emergence was evaluated in the presence or not of GSK343 or DZNepA as indicated after 10 days. The quantification of emergence is presented (*n* = 5; *p < 0.5; **p < 0.01, ***p < 0.001). **b**,** c** LS174T cells have been transfected with siRNAs directed against EZH2 after 4 days of treatment. EZH2 expression has been detected by western blot (*n* = 4) and emergence was evaluated after 10 days (*n* = 13; ****p < 0.0001). **d**, **e** LS174T cells have been transfected with siRNAs directed against SUZ12 after 4 days of treatment. SUZ12 expression has been detected by western blot (*n* = 4) and emergence was evaluated after 10 days (*n* = 3). **f** PLC clones were generated as described above, emergent cells were then trypsinized and further treated with GSK343 (5 µM) and/or Palbociclib (0.5 µM) for 2 days. The percentage of senescent cells was evaluated as the number of cells expressing SA-β-gal activity (*n* = 5). Proliferation of PLC was evaluated by flow cytometry using an antibody directed against the Ki67 antigen (*n* = 5). DNA DAPI staining was used to evaluate the percentage of cells in SubG1 phase by flow cytometry (*n* = 5)

To determine if EZH2 was involved in cell emergence, we first used two different inhibitors: 3-deazaneplanocin A (DZNep), which is widely used to downregulate EZH2 levels, and GSK343, which is more specific and targets the enzymatic activity of the protein. LS174T cells were treated with sn38 for 4 days and emergent cells were then generated in the presence or absence of increasing concentrations of the two drugs (Fig. 4a). Results showed that EZH2 inhibition significantly reduced the number of emergent cells and this effect was observed with both inhibitors. As a control, ABT737 which targets the Bcl-2 and Bcl-xL prosurvival proteins had no effect (data not shown). This inhibition of emergence was then confirmed using RNA interference. Western blot experiments confirmed that the methylase was downregulated following siRNA transfection (Fig. 4b). Results showed that EZH2 inactivation significantly reduced senescence escape (Fig. 4c). To determine if other members of the PRC2 complex were involved in cell emergence, we inactivated the expression of the SUZ12 protein. Western blot experiments confirmed its downregulation (Fig. 4d), but no significant effect was observed on CIS escape (Fig. 4e). This result suggested that the effect of EZH2 might not be related to the transcriptional repression mediated by the PRC2 complex.

If the cdk4–EZH2 pathway is necessary to induce senescence escape, we reasoned that these proteins might be targeted to reduce the proliferation of dividing clones that have resisted to the treatment. To test this hypothesis, we generated emergent cells for 10 days, trypsinized the cells and regrew them for 2 days in the presence or absence of the two inhibitors. After 10 days of emergence, the proportion of dividing clones increased significantly so that around 70% of cells proliferated as shown by the expression of the Ki67 antigen after 2 days of culture (Fig. 4f, lane 1). The inhibition of cdk4 or EZH2 reduced proliferation and this was more significant when palbociclib and GSK343 were added together (Fig. 4f, compare lanes 4 and 2–3). Using β-galactosidase staining, we observed that palbociclib induced a significant increase in the number of senescent cells and this was not significantly enhanced in the presence of GSK343 (Fig. 4f, compare lanes 5, 7 and 8). In addition, no subG1 cells were detected in these conditions, suggesting that apoptosis was not induced and that palbociclib and GSK343 essentially acted at the level of cell proliferation and senescence.

Overall, we concluded from these results that EZH2 was necessary for cell emergence in response to sn38.

### The EZH2–AP2M1 pathway regulates senescence escape

**Fig. 5:**
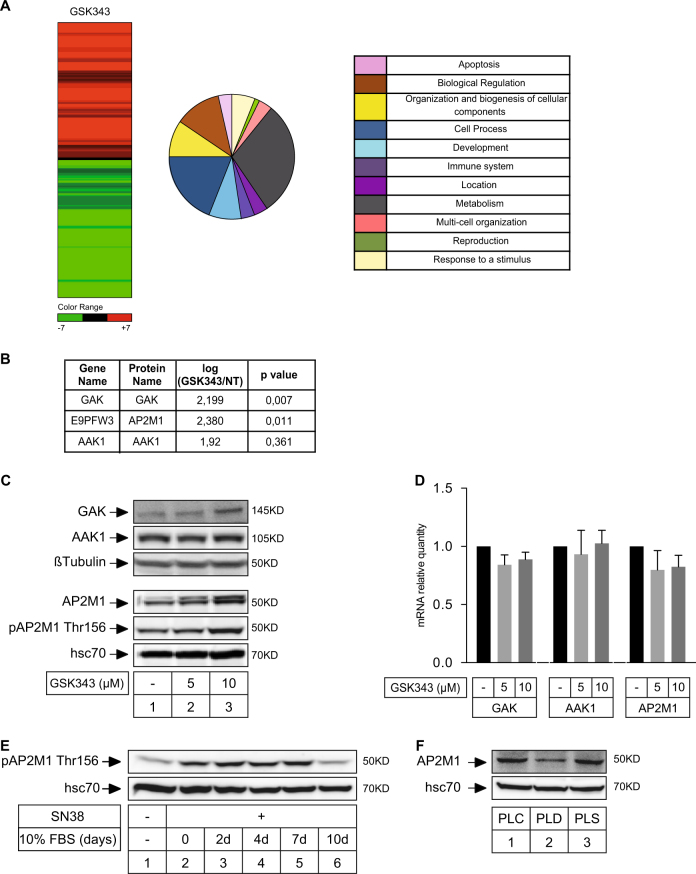
Quantitative proteomic analysis of EZH2 targets. **a** Quantitative mass spectrometry analysis of cells treated with GSK343 (10 µM) for 48 h (*n* = 3). A heat map presents the relative proportion of downregulated and upregulated proteins. Upregulated proteins have been classified according to different biologic pathways using the PATTERN software. See also supplementary Table 1. **b** Relative quantification and significance of GAK, AAK1 and AP2M1 expressions obtained by mass spectrometry analysis. **c** Western blot analysis of the expression of GAK, AAK1, AP2M1 and of its Thr 156 phosphorylated form following GSK343 treatment (*n* = 4). **d** Analysis of GAK, AAK1 and AP2M1 mRNA expressions by RT-QPCR (*n* = 3). **e** LS174T cells have been stimulated with sn38 for 96 h and emergence was induced after 4 days by serum stimulation. AP2M1 expression was evaluated by western blot analysis at the indicated time (*n* = 3). **f** Emergent cells were sorted by flow cytometry after 7 days according to low (PLD) or high (PLS) FSC/SSC values. AP2M1 expression was evaluated by western blot analysis (*n* = 6), either in the total emergent population (PLC), in the senescent cells (PLS) or in dividing clones (PLD)

**Fig. 6:**
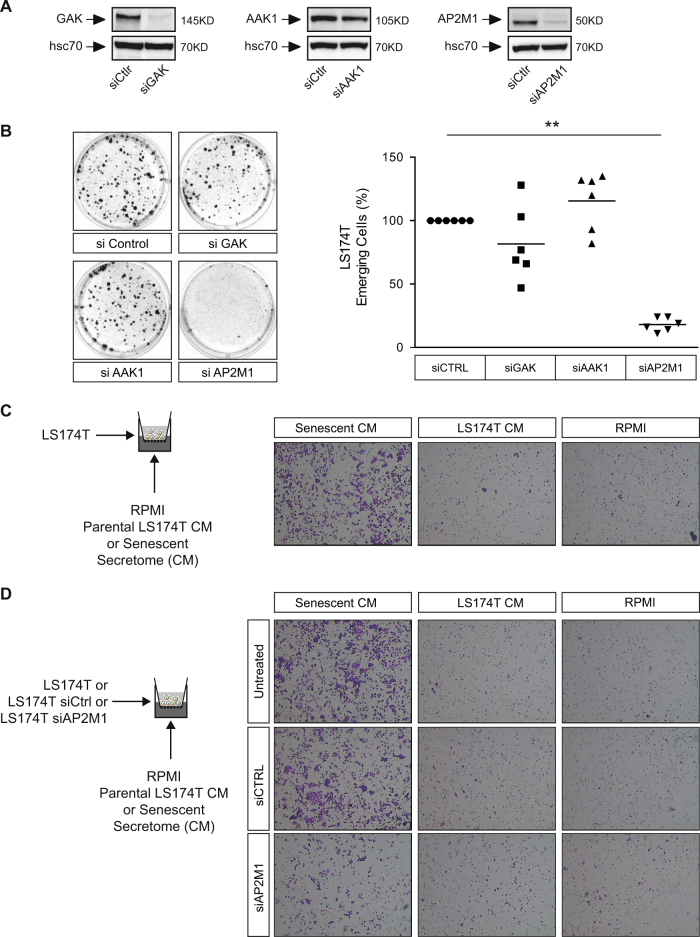
AP2M1 regulates CIS escape and is involved in the transmission of soluble signals from senescent cells. **a** LS174T cells have been transfected with the corresponding siRNAs and protein expression was analyzed by western blot (*n* = 3). **b** Following treatment and siRNA transfection, emergence was evaluated after 10 days. Representative images are shown on the left part of the figure and the quantification of emergent clones is presented on the right part (*n* = 6; **p < 0.01). **c** Conditioned media (CM) from parental or senescent cells were collected after 24 h of serum starvation. Migration assays were performed using Boyden inserts. RPMI or conditioned media supplemented with 3% FBS were deposited at the bottom of the well, parental LS174T cells at the top. After 72 h, migrating cells were stained with crystal violet (*n* = 3). **d** RPMI or conditioned media collected as above supplemented with 3% FBS were deposited at the bottom of the the well. Parental LS174T cells or cells transfected with the indicated siRNAs were deposited at the top to evaluate the sensitivity of cells expressing or not AP2M1 to soluble signals generated from senescent cells. After 72 h, migrating cells were stained with crystal violet (*n* = 3)

We then used a quantitative proteomic analysis to identify potential targets regulated by EZH2 and involved in senescence escape. To this end, LS174T cells were stimulated or not with GSK343 for 48 h, the experiment was repeated three times and protein signatures were identified as previously described (see Materials and Methods and refs. ^17,18^). In response to EZH2 inhibition, 104 proteins were significantly deregulated (*p*-value > 0.05), 57 were upregulated and 47 downregulated (Fig. 5a and Supplementary Table 1). Among these proteins, we focused on AP2M1, GAK and AAK1 that were upregulated in response to GSK343 (Fig. 5b, note that the *p*-value was not significant for AAK1). During senescence, arrested cells produce specific soluble factors known as the senescence-associated secretory phenotype (SASP)^19–21^. This secretome plays an important role in tumor suppression but recent results have shown that its outcome is complex since these secreted proteins have also oncogenic functions^22–24^. In our experimental conditions, we have effectively observed that senescence escape is regulated by secreted proteins that induce emergence, cell migration and anoikis resistance (Moreau et al. submitted, see below Fig. 6). Although it is clear that the SASP plays an essential role during senescence, much less is known about the cell surface receptors that transmit the corresponding signaling pathways. Since AP2M1, GAK and AAK1 are involved in receptor endocytosis^10^, this suggested to us that these proteins could mediate some effects of the SASP. Western blot analysis indicated that the expression of AAK1 was not significantly modified following EZH2 inhibition but that GAK and AP2M1 were upregulated. Accordingly, the active phosphorylated form of AP2M1 was also increased (Fig. 5c). No modifications of these proteins were observed at the mRNA level (Fig. 5d), further suggesting as recently described^25^ that the function of EZH2 does not always rely on the transcriptional repression mediated by the PRC2 components. Using kinetic experiments, we observed that AP2M1 was upregulated during the initial step of senescence escape (Fig. 5e, lanes 3–5). After 10 days, its expression decreased back to control level, certainly as a consequence of the emergence of dividing clones that reexpressed EZH2. Accordingly, using cell sorting we observed after 7 days that AP2M1 was upregulated in the PLS senescent population and downregulated in PLD dividing cells (Fig. 5f, lanes 2–3). To determine if these proteins were involved in senescence escape, their expression was inactivated by RNA interference and the downregulation was verified by western blot (Fig. 6a). Interestingly, whereas GAK or AAK1 had no significant effect on cell emergence, the inactivation of AP2M1 almost completely blocked CIS escape (Fig. 6b).

We then determined if AP2M1 was involved in the transmission of a secreted signal. To this end, we used conditioned media (CM) obtained from senescent or non-treated cells. We tested their influence on cell migration as an indicator of increased transformation and of a deleterious effect of senescent cells. As previously reported^20^, the secretome produced by senescent cells significantly enhanced cell migration as compared to the CM obtained from parental LS174T cells (Fig. 6c). To determine if AP2M1 was involved in this mechanism, its expression was downregulated by RNA interference and the corresponding cells were transferred to the top of the boyden chambers. The attractive effect of the secretome of senescent cells was then tested on cells that expressed or not AP2M1. When control siRNA were used, no modification of cell migration was observed. Interestingly, the downregulation of AP2M1 significantly reduced cell invasion (Fig. 6d).

Altogether, we concluded from these results that AP2M1 is a target of EZH2 involved in the control of CIS escape and in the transmission of soluble signals produced by senescent cells.

### Regulation of AP2M1 during apoptosis

**Fig. 7:**
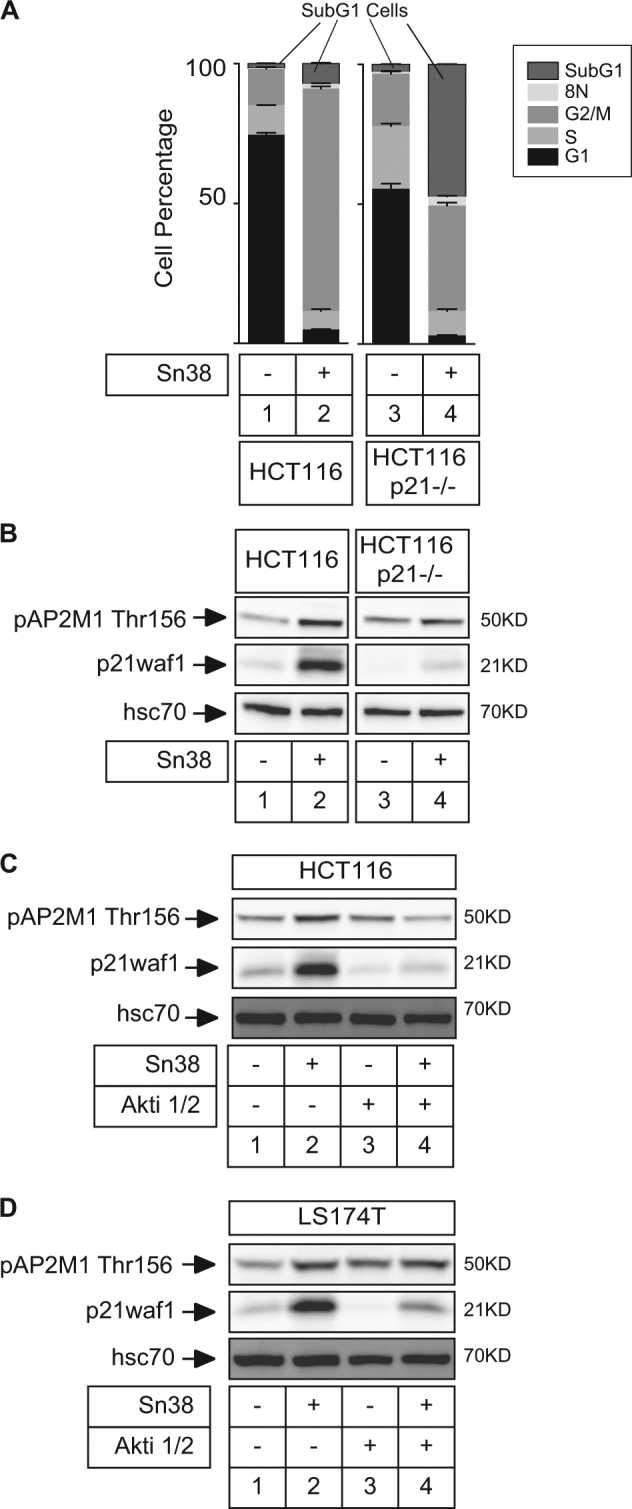
AP2M1 expression following apoptosis induction. **a** HCT116 and HCT116 p21−/− cells have been treated with sn38 for 72 h (5 ng/ml) and apoptosis was evaluated by FACS analysis and the detection of subG1 cells (*n* = 3 ± s.d.). **b** Phospho-AP2M1 and p21waf1 expressions have been analyzed by western blot in the indicated cells (*n* = 3). **c**,** d** LS174T or HCT116 cells were treated with sn38 in the presence or absence of Akti 1/2 for 72 h (10 µM) and phospho-AP2M1 and p21waf1 expressions were analyzed as described above (*n* = 3)

Besides senescence, apoptosis also plays an essential role in tumor suppression. We have recently shown that sn38 induced cell death instead of senescence when p21waf1 is not expressed, either in HCT116 p21−/− cells or when added in combination with an Akt inhibitor in LS174T cells^7^. We therefore examined the expression of AP2M1 during apoptosis induction. We first used HCT116 p21–/– cells since they are more sensitive to cell death and apoptosis was verified by the detection of subG1 cells following sn38 treatment (Fig. 7a). Results showed that AP2M1 upregulation was limited in the absence of the cell cycle inhibitor (Fig. 7b, compare lanes 1–2 and 3–4). When apoptosis was induced in HCT116 cells by Akt inhibition (see ref. ^7^), results also showed that AP2M1 was not significantly upregulated (Fig. 7c, compare lanes 4 and 2). However, when this experiment was repeated in LS174T cells, we found that the induction of AP2M1 was not modified by Akt inhibition and cell death induction (Fig. 7d, lanes 4 and 2). In the two cell lines, p21waf1 induction was reduced as expected following inhibition of the kinase.

Altogether, we concluded from these results that AP2M1 expression varies between HCT116 and LS174T cells during the induction of apoptosis.

## Discussion

The definition of a tumor suppressive mechanism implies that it has to be inactivated during tumor progression. When tested experimentally, this definition therefore depends on the experimental model. A definitive arrest might be specific to primary cells and as such amounts to a situation that represents the early step of carcinogenesis. This is not really relevant for cancer treatment since chemotherapy does not concern primary cells but clones that have inactivated their suppressive pathways. This is illustrated by cell lines that do not express p16INK4 anymore and for this reason have a reduced senescence capacity. Cancer cell lines that have retained at least some aspects of senescence are probably more representative of the tumors and relapses faced by oncologists. Therefore, in the therapeutic context of cancer treatment, it is more difficult to say that chemotherapy-mediated senescence is always irreversible since by definition its regulation has to be inactivated to some extent. As stated above, it has been shown recently that an established senescence can be reversed in melanocytes following PTEN depletion and in keratinocytes^4,26,27^. Equivalent adaptations have been described in bacteria where persistent subclones can reconstitute a total population following antibiotic treatment^28^. In our model we therefore favored the hypothesis that CIS is not complete and that a phenotypic switch allows persistence.

In this work, we pursued our experiments on CIS escape, showing that the cdk4 kinase is involved in cell emergence. As previously reported^29,30^, we also observed that cyclin D1 was upregulated early during CIS escape. It is not really surprising that the cdk4–E2F pathway is involved in the proliferation of PLD subclones and we can speculate that its classical cell cycle targets are involved in emergence. Among these, it is interesting to note the importance of the EZH2 methylase. This protein is overexpressed in several types of tumors, and it has been described as an important target of the E2F pathway and as a new therapeutic target in lymphoma^14,31^. Importantly, EZH2 is involved in the transcriptional repression of the INK4 locus^15^. During senescence induction, the methylase is inhibited to allow p16INK4 upregulation in cooperation with the JMJD3 demethylase^32,33^. Although we did not observe any induction of p16, it will be interesting to determine if JMJD3 is involved in our model of CIS escape. The inability to upregulate p16INK4, maybe through an imperfect demethylation, might be one reason that explains CIS emergence.

Through proteomic analysis, we identified new potential targets of EZH2 among which AP2M1 appears to play an important role in CIS escape. It is striking to note that this protein plays an essential role in receptor endocytosis^10^. Since senescent cells are well known to produce secreted proteins, this implies that specific cell surface receptors are involved in the regulation of senescence pathways. We speculate that AP2M1 regulates the corresponding receptors and further experiments will clarify its role and targets and investigate which cells express them, the dividing or senescent subpopulations. This led to the hypothesis that senescent cells might express specific receptors that could be useful, either to identify these arrested cells or to be used as new therapeutic targets.

Altogether, these results indicate that subpopulations of cells can escape senescence with the help of arrested cells and through the activation of the cdk4–EZH2 pathway. Since drugs targeting cdk4 and EZH2 are entering clinical trials, we propose that they should be tested in the treatment of cancers that become resistant to first-line genotoxic therapies.

## Materials and methods

### Cell lines and emergence

Cell lines (American Type Culture Collection) were maintained in antibiotic-free RPMI-1640 medium (Lonza), supplemented with 10% FBS and maintained at 37 °C in 5% carbon dioxide. Cells were routinely tested for the absence of mycoplasma contamination. For persistent cell generation, 50,000 cells in 6-well plates were treated for 96 h with sn38 (5 ng/ml; Tocris Bioscience 2684) or Doxorubicin (15 ng/ml, Tocris Bioscience 2252) in 2 ml 3% FBS, washed with phosphate-buffered saline (PBS) and then restimulated with fresh 10% FBS for 7 to 10 days. The other treatments were used in the same conditions: Palbociclib (PD0332991; Sigma Aldrich), GSK343 (0.1–10 µM; Sigma Aldrich), DZnepA (0.005–0.5 µM; Sigma Aldrich) and ABT737 (0.005–0.5 µM; Selleckchem).

### Cell transfection, siRNA

Cells were cultured for 24 h in 6-well plates (200,000 cells) and transfected with the following siRNAs (50 nM): Cdk4 (Dharmacon; L-003238-00-0005), EZH2 (Dharmacon; L-004218-00-0005), SUZ12 (Dharmacon; L-006957-00-0005), GAK (Dharmacon; L-005005-00-0005), AAK1 (Dharmacon; L-005300-02-0005), AP2M1 (Dharmacon; L-008170-00-0005) or control siRNA (Dharmacon; D-001810-10-05), using DharmaFect-4 according to the manufacturer’s instructions.

### β-Galactosidase staining

Cells (50,000 cells/6-well plates for 4 days of treatment) were fixed for 15 min (room temperature) in 2% formaldehyde, washed with PBS and incubated at 37 °C with fresh staining solution: 0.3 mg/ml of 5-bromo4-chloro-3-indolyl β-d-galactoside (X-Gal, Fermentas), 40 mM citric acid (Sigma), 40 mM sodium phosphate (Sigma; stock solution (400 mM citric acid, 400 mM sodium phosphate) must be at pH 6), 5 mM potassium ferrocyanide (Sigma), 5 mM potassium ferricyanide (Sigma), 150 mM NaCl (Sigma) and 150 mM MgCl_2_ (Sigma). The senescence-associated (SA)-β-Gal-positive cells were quantified after 16–20 h as compared to unstained cells.

### Western blotting

The following antibodies were used: rabbit monoclonal anti-p21 (1:1000; Cell Signaling 2947), rabbit polyclonal anti-cdk4 (1/1000; Santa Cruz sc 260), rabbit polyclonal anti-cyclin D1 (1/1000; Santa Cruz sc 753), mouse monoclonal anti-HSC70 (1/1000, Santa Cruz sc 7298), rabbit monoclonal anti-EZH2 (1/1000; Cell Signaling 5246), mouse monoclonal anti-Rb (pS780) (1/1000; BD Pharmingen 558385), mouse monoclonal anti-AAK1 (1/750; R&D System MAB6886), mouse monoclonal anti-GAK (1/1000; R&D System MAB6918), mouse monoclonal anti-AP2M1 (1/1000; NovusBio NBP2-00834), rabbit polyclonal anti-AP2M1 (Thr 156) (1/1000; Cell Signaling 7399) and mouse monoclonal anti-SUZ12 (1/1000; R&D System MAB4184).

### Quantitative PCR

Analysis was performed using the comparative CT method (2^(ΔCt)), according to the endogenous housekeeping gene RPLPO or G3PDH. PCR primers sequences were as follows: RPLPO (5′-AACCCAGCTCTGGAGAAACT-3′ and 5′-CCCCTGGAGATTTTAGTGGT-3′), G3PDH (5′-GAAGGTGAAGGTCGGAGTC-3′ and 5-GAAGATGGTGATGGGATTTC-3′), EZH2 (5′-AGGAGTTTGCTGCTGCTCTC-3′ and 5′-CCGAGAATTTGCTTCAGAGG-3′), GAK (5′-CGTGCGACACGGTTCTGAA-3′ and 3′-TGGTCCCTTGGTTACTAAGCAA-5′), AP2M1 (5′-ACGTTAAGCGGTCCAACATTT-3′ and 5′-GCCATCACGTCACACATCTTAT-3′), AAK1(5′-AGTGGCTACATCGGAAGAGTC-3′ and 5′-AGGCACATTTCATCCCATTGC-3′), CDKN1A (5′-GCTCCTTCCCATCGCTGTCA-3′ and 5′-TCACCCTGCCCAACCTTAGA-3′).

### Immunofluorescence

Cells were fixed with 2% formaldehyde solution for 15 min at room temperature and then permeabilized in ethanol 70% overnight at 4 °C. After 3 washing with PBS–Tween 0.02%, cells were saturated in PBS–BSA 2% for 10 min at room temperature. During 4 h, cells were incubated with the following primary antibodies (1/100e): mouse control IgG (Cell Signaling, 3900S), rabbit control IgG (Cell Signaling, 3900S), rabbit anti-EZH2 IgG (Cell Signaling) and mouse anti-Ki67 IgG1 (Cell Signaling, 9449S). After 3 washing with PBS–Tween 0.02%, cells were saturated in PBS–BSA 2% for 10 min at room temperature. Cells were incubated with the following secondary antibodies (1/200e): goat anti-Mouse IgG Alexa 488 (Invitrogen, A11001) or Goat anti-Rabbit IgG secondary antibody Alexa 568 (Invitrogen, A11011). Cells were washed 3 times with PBS–Tween 0.02% and covered with Prolong Diamond with 4',6-diamidino-2-phenylindole (DAPI).

### Mass spectrometry analysis

#### Creation of the spectral library

In order to build the spectral library, peptide solutions of several protein samples were analyzed by a shotgun approach by micro liquid chromatography–tandem mass spectrometry (micro-LC–MS/MS). Five pooled samples of breast, colorectal and blood tissues were prepared to obtain a good representation of the peptides. Each sample were fractionated by offgel fractionator in 24 fractions. Each fraction was separated into a micro-LC system Ekspert nLC400 (Eksigent, Dublin, CA, USA) using a ChromXP C18CL column (0.3 mm × 15 cm, 3 μm, 120 Å) (Eksigent) at a flow rate of 5 μl/min. Water and acetonitrile (ACN), both containing 0.1% formic acid, were used as solvents A and B, respectively. The following gradient of solvent B was used: 0 to 5 min 5% B, 5 to 125 min 5 to 35% B, then 9 min at 95% B, and finally 9 min at 5% B for column equilibration. As the peptides eluted, they were directly injected into a hybrid quadrupole time-of-flight (TOF) mass spectrometer Triple TOF 5600+ (Sciex, Redwood City, CA, USA) operated with a ‘top 30′ data-dependent acquisition system using positive ion mode. The acquisition mode consisted of a 250 ms survey MS scan from 400 to 1250 *m/z*, followed by an MS/MS scan from 200 to 1500 *m/z* (75 ms acquisition time, 350 mDa mass tolerance, rolling collision energy) of the top 30 precursor ions from the survey scan.

Peptide and protein identifications were performed using Protein Pilot software (version 5.0, Sciex) with a human Swiss-Prot/TrEMBL concatenated target-reverse decoy database (downloaded in March 2016) containing 142,441 target human protein sequences, specifying MMTS as Cys alkylation. The false discovery rate (FDR) was set to 0.01 for both peptides and proteins. The MS/MS spectra of the identified peptides were then used to generate the spectral library for SWATH peak extraction using the add-in for PeakView Software (version 2.2, Sciex) MS/MS^ALL^ with SWATH Acquisition MicroApp (version 2.0, Sciex). Peptides with a confidence score above 99% as obtained from Protein Pilot database search were included in the spectral library.

#### Relative quantification by SWATH acquisition

Samples were analyzed using a data independent analysis (DIA) method. Each sample (5 μg) was analyzed using the LC–MS equipment and LC gradient described above, using a SWATH-MS acquisition method. The method consisted of repeating the whole gradient cycle, which consisted of the acquisition of 35 TOF MS/MS scans of overlapping sequential precursor isolation windows (25 *m/z* isolation width, 1 *m/z* overlap, high sensitivity mode) covering the 400 to 1250 *m/z* mass range, with a previous MS scan for each cycle. The accumulation time was 50 ms for the MS scan (from 400 to 1250 *m/z*) and 100 ms for the product ion scan (230 to 1500 *m/z*), thus making a 3.5 s total cycle time.

#### Data analysis

The targeted data extraction of the SWATH runs was performed by PeakView using the MS/MS^ALL^ with SWATH Acquisition MicroApp. PeakView processed the data using the spectral library created from the shotgun data. Up to 10 peptides per protein and 7 fragments per peptide were selected, based on signal intensity; any shared and modified peptides were excluded from the extraction. The retention times from the peptides that were selected for each protein were realigned in each run according to iRT peptides (Biognosys AG, Schlieren/Zürich, Switzerland) spiked in each sample and eluting along the whole time axis; the extracted ion chromatograms were generated for each selected fragment ion. PeakView computed a score and an FDR for each assigned peptide using chromatographic and spectra components; only peptides with an FDR of less than 5% were used for protein quantitation. The peak areas for peptides were obtained by summing the peak areas of the corresponding fragment ions; protein quantitation was calculated by summing the peak areas of the corresponding peptides. MarkerView (version 1.2, Sciex) was used for signal normalization, and differential abundance was tested by applying a *t*-test at the protein level.

### Statistical analysis

Numerical data are presented as mean±s.d. or mean of clone number and value of each replicate. A significant difference was analyzed by non parametrics tests (test-*t* and Mann–Whitney) and considered when the *p*- value was less than 0.05 and was represented by *p < 0.05, **p < 0.01, ***p < 0.001 and ****p < 0.0001.

## Electronic supplementary material

Supplementary Figure Legend

Supplementary Methods

Supplementary Figure 1

Supplementary Figure 2

Supplementary Table 1
