# Supplementary material for: Regulation of senescence escape by the cdk4–EZH2–AP2M1 pathway in response to chemotherapy
Source: Cell Death Dis. 2018 Feb 7;9(2):199. doi: 10.1038/s41419-017-0209-y (PMC5833455; doi:10.1038/s41419-017-0209-y)
Supplement: Supplementary file 1 — Supplementary Figure Legend [file 41419_2017_209_MOESM1_ESM.doc]

**SUPPLEMENTARY FIGURES LEGEND**

**Supplementary Figure 1: Pre-existent clones are not selected during the treatment.**

**A.** LS174T cells have been stimulated or not with sn38 (5ng/ml) for 4 days as indicated in the presence of 3% FBS. In parallel, quiescent cells were generated for 4 days in the absence of serum. Cells were then stimulated with 10% FBS for 2 days.

**B.** Analysis of cyclin A expression by western blot following serum stimulation of quiescent or senescent cells (n=6).

**C.** Analysis of mcm2, mcm3 and cdc25A mRNAs expression by RT-QPCR following serum stimulation of quiescent or senescent cells (n=4, each gene was normalized according to the values obtained following sn38 treatment, bars #4).

**D.** Cells seeded in 96 cells at the concentration of 4 cells/well were grown in 3% FBS or in 3%FBS plus sn38 (5ng/ml). The number of dividing clones was evaluated after 4 days.

**Supplementary Figure 2: Chromatin Immunoprecipitation analysis of the cyclin D1 promoter**

**A.** LS174T cells have been stimulated with Sn38 (5ng/mL) for 72 hours and the expression of the cyclin D1 mRNA was evaluated by quantitative RT-PCR (n=4 +/- sd).

**B.** Representation of the analyzed regions of the cyclin D1 promoter.

**C.** LS174T cells have been stimulated with Sn38 (5ng/mL) for 72 hours. DNA was amplified using pair of primers that covers the indicated regions following immunoprecipitation of the EGFR, E2F1, type II RNA polymerase or control antibodies as indicated (n=3 +/- sd).

**D.** As a control, the recruitement of type II RNA polymerase and E2F1 was analyzed on the proxymal p21waf1 promoter and compared to the signal obtained with a control antibody (n=3 +/- sd).
