# Supplementary material for: Regulation of senescence escape by the cdk4–EZH2–AP2M1 pathway in response to chemotherapy
Source: Cell Death Dis. 2018 Feb 7;9(2):199. doi: 10.1038/s41419-017-0209-y (PMC5833455; doi:10.1038/s41419-017-0209-y)
Supplement: Supplementary file 2 — Supplementary Methods [file 41419_2017_209_MOESM2_ESM.doc]

**SUPPLEMENTARY Materials and Methods**

**Secretome Production**. Following senescence induction for 4 days, Ccells were starved for 24 hours with RPMI media 0% FBS. Supernatants were harvested and to eliminate cell debris, centrifuged at 200g for 5 minutes.

**Migration assay in Boyden Chambers.** Cells were harvested and resuspended in serum-free culture medium. 40000 cells were added in suspension in 200 μl in each insert. Below the inserts, 700 μl of RPMI or of secretome complemented with 3% FBS were added and cells were incubated for 72 hours. Cells were then fixed, washed with PBS and stained with crystal violet.

**Flow Cytometry**. **Ki67 staining –** Trypsinized cells were washed with PBS and incubated with cold 70% methanol at 4°C for 30 min. Cells were then washed twice with PBS 0.1% Tween and PBS 0.1% Tween 1% BSA. 250 000 cells were then incubated for 30 min at room temperature with FITC mouse anti-human Ki-67 or with FITC mouse IgG1 as control isotype (BD Pharmingen kit 556026).

**Cell cycle analysis by the DNA Vindelov 83 approach** – Trypsinized cells were washed with PBS. 150 000 cells were incubated 10 minutes in the dark at RT with 300µL of solution A (trypsin 30 μg/mL, Sigma). 150µL of Solution B (trypsin inhibitor 0,5 mg/mL, RNAse A 0.1 mg/mL, Sigma) was then added for 10 minutes in the dark at RT. Then, solution C (propidium iodide 0.6 mM, spermine tetrahydrochloride 3.3 mM, Sigma) was added for 10 minutes in the dark at 4°C. Solutions were prepared in a storage buffer pH 7.6 containing 3.4 mM sodium citrate 2H20 (Sigma), 0.1% Igepal CA-630 (Sigma), 3 mM spermine tetrahydrochloride and 1mM tris-aminomethane. Cells were then washed and resuspended in PBS before analysing.

**Chromatin Immunoprecipitation (ChIP) assay.** LS174T cells were cross-linked with 1% formaldehyde for 10 minutes at room temperature. Cross-linking was stopped by adding 0.125 mol/L glycine for 5 min. Cells were washed 3 times with cold phosphate-buffered saline (PBS). Cells were then scraped and washed three more times with cold PBS. Pellets were resuspended in 1mL of lysis buffer (5 mM PIPES, 85 mM KCl, 0,5% NP40). All buffers were supplemented with proteases and phosphatases inhibitors (1 mM PMSF, 10 μg/ml aprotinin, 10 μg/ml leupeptin, 10 μg/ml pepstatin, 1 mM Na3VO4, 50 mM NaF). Samples were incubated for 15 min at 4°C and vortexed for 30 seconds every 2 min. Cells were centrifuged 10 min, 12,000rpm at 4°C. Supernatants were discarded and pellets were resuspended in 500µL sonicating buffer (10 mM EDTA, 1% SDS, 50mM Tris-EDTA pH=8). Nuclei extracts were sonicated (4 cycles of 25 seconds of sonication and 25 seconds in the ice) to obtain DNA fragments of about 500 to 1,000 pb. Supernatants were recovered by centrifugation at 12,000 rpm for 10 min at 4 °C and diluted ten times with IP buffer (0,01% SDS, 1.1% Triton X-100, 1.2 mM EDTA, 16.7 mM Tris-HCl (pH = 8.0), 167 mM NaCl).

# For each 500 μL extract, 5µL DTT (0,1M), 15μl of ChIP-grade Protein A/G Magnetic beads (ThermoFisher), beforehand conjugated with 2 μg specific antibodies were added. After incubation overnight at 4°C, beads were washed with 600µL TSE1 buffer (1% Triton X-100, 150mM NaCl, 20mM Tris-HCl pH8,1, 0,1% SDS, 2mM EDTA), 600µL TSE2 buffer (1% Triton X-100, 500mM NaCl, 20mM Tris-HCl pH8,1, 0,1% SDS, 2mM EDTA) and 600µL TSE3 buffer (1% NP40, 1% Sodium deoxycholate, 250mM LiCl, 10mM Tris-HCl pH8,1). Following two washes in TE buffer (10mM Tris-HCl, 1mM EDTA), samples were eluted with 200µL of freshly elution buffer (1% SDS, 0,1M NaHCO_3_). Crosslink was reversed by adding 8µL NaCl (5M) and 2µL EDTA (0,5M) to the samples and incubating overnight at 65°C.

DNA was purificated using a High Pure PCR Template Preparation Kit (Roche) and analysed by Q-PCR.

The following antibodies have been used: rabbit polyclonal anti-EZH2 (Cell Signaling 5246), rabbit polyclonal anti-E2F1 (Santa Cruz sc 193), rabbit polyclonal anti-POLII (Santa Cruz sc 899), rabbit polyclonal anti-EGFR (Santa Cruz sc 03), rabbit mAb IgG Isotype Control (Cell Signaling 39005).

The regions were amplified using the following primers: Cyclin D1 promoter -521/-218 (5’ ATTCTTTGGCCGTCTGTCCG 3’ and 5’ GCCCCTGTAGTCCGGTTTTC 3’) ; Cyclin D1 promoter -757/-218 (5’ CTGCTGCTGGAATTTTCGGG 3’ and 5’ TATCCAAGCCGGCAGAATGG 3’) ; Cyclin D1 promoter -1029/-691 (5’ AACCTTCGGTGGTCTTGTCC 3’ and 5’ TTTCAGCTTAGCATGCGCTC 3’) ; Cyclin D1 promoter - 86/+16 (5’ CCGGGCTTTGATCTTTGCT 3’ and 5’ GACTCTGCTGCTCGCTGCTA 3’). Cyclin D1 Exon 2 +1921/+2035 (5’ GAACTACCTGGACCGCTTCC 3’ and 5’ TAGATGCACAGCTTCTCGGC 3’) ; Cyclin D1 Intron 2 +2470/+2524 (5’ GATCTGGGATTGCGTGTTGC 3’ and 5’ TCCTGGATGTTGGAGGGGAG 3’) ; Cyclin D1 Exon5 +10653/+10793 (5’ GCTGTAGTGGGGTTCTAGGC 3’ and 5’ GGCACGCTACGCTACTGTAA 3’) ; EZH2 promoter -979/-601 (5’ CGGCGGTTAAAACCGTTACC 3’ and 5’ GGGTGCTGGAAGCCAAGTTT 3’) ; EZH2 promoter -1034/-959 (5’ GCGATAAGCACTGCAAGCTC

3’ and 5’ TATGTCTGCTGCTGCCTTGG 3’) ; EZH2 promoter -495/-452 (5’ CGGCGGTTAAAACCGTTACC 3’ and 5’ GGGTGCTGGAAGCCAAGTTT3’) ;  p21 promoter -84/+4 (5’ GCGGCGCGGTGGGCCGAGCGCGGG 3’ and 5’ GGCTCCACAAGGAACTGACT 3’).
