# Supplementary figures and images for: Regulation of senescence escape by the cdk4–EZH2–AP2M1 pathway in response to chemotherapy
Source: Cell Death Dis. 2018 Feb 7;9(2):199. doi: 10.1038/s41419-017-0209-y (PMC5833455; doi:10.1038/s41419-017-0209-y)

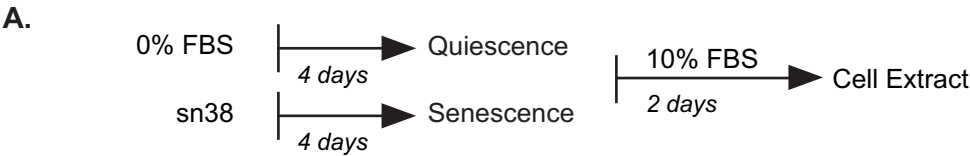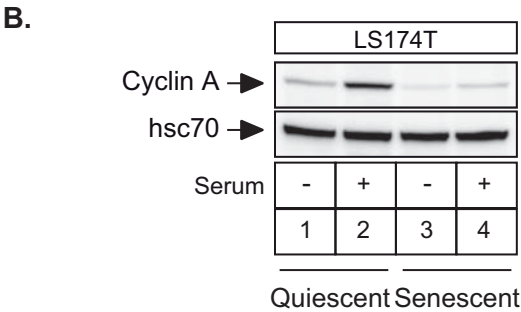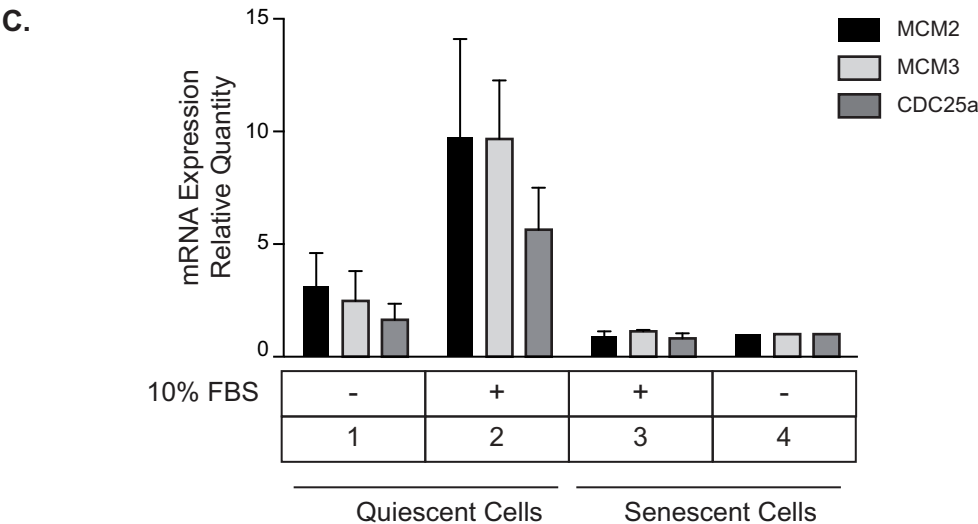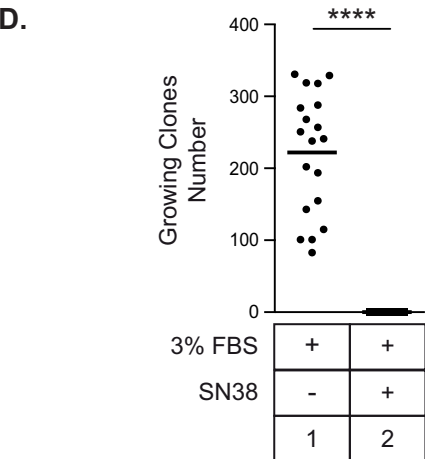

Supplement: Supplementary file 3 — Supplementary Figure 1 [file 41419_2017_209_MOESM3_ESM.pdf]

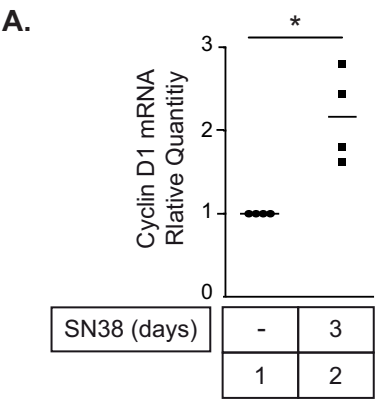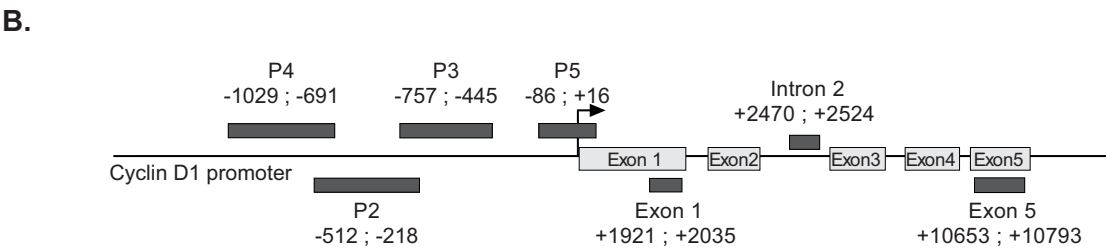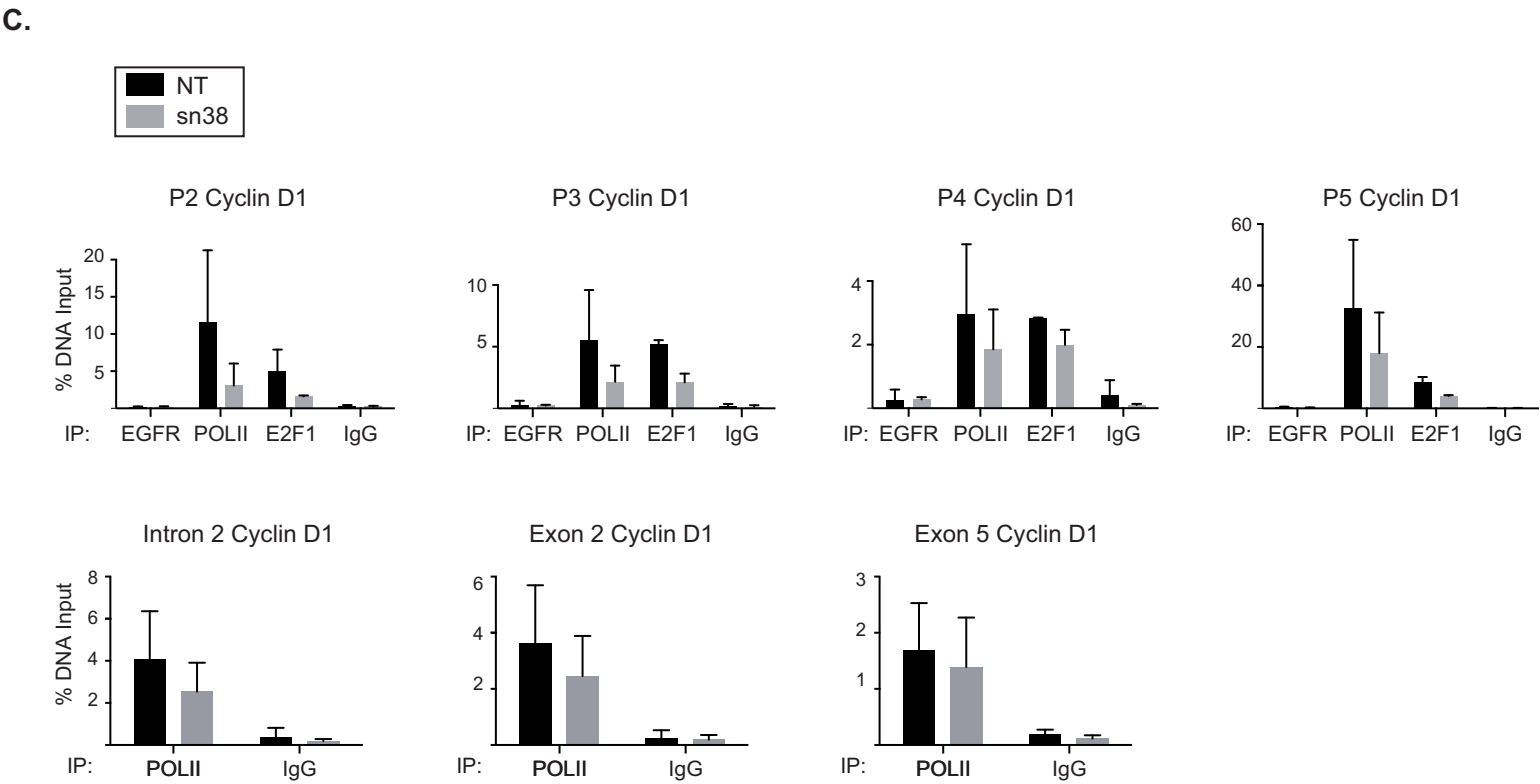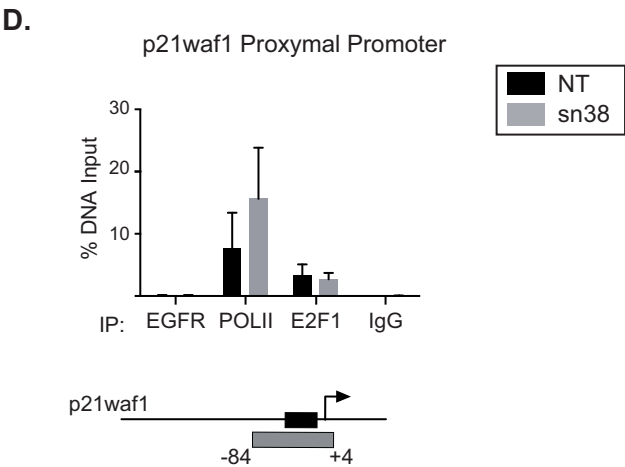

Supplement: Supplementary file 4 — Supplementary Figure 2 [file 41419_2017_209_MOESM4_ESM.pdf]
